# Open-Air Plasma Treatment to Enhance Nanoparticle Adhesion on Nylon- and Cotton-Based Fabrics for Chemical Protection

**DOI:** 10.1021/acsami.5c20116

**Published:** 2026-01-02

**Authors:** Saurabh Karande, Owen C. Grimm, Natalie L. Pomerantz, Gregory W. Peterson, James E. Whitten

**Affiliations:** † Department of Chemistry, 14710 University of Massachusetts Lowell, Lowell, Massachusetts MA 01854, United States; ‡ US Army Combat Capabilities Development Command Soldier Center, Natick, Massachusetts 01760, United States; § US Army Combat Capabilities Development Command Chemical Biological Center, Aberdeen Proving Ground, Maryland 21010, United States

**Keywords:** Adhesion, Metal−Organic Frameworks, Metal Oxide Nanoparticles, Plasma Treatment, X-ray Photoelectron Spectroscopy, Zinc Oxide

## Abstract

Nylon- and cotton-based fabrics, particularly a 50:50 nylon-cotton blend (“NYCO”), are commonly used for military and workforce garments. Developing high-throughput methods to attach reactive or sorptive metal oxide or metal–organic framework (MOF) nanoparticles (NPs) is important for chemical protection. Open-air plasma treatment has been used to activate cotton, nylon, and NYCO such that they covalently bond to ca. 20 nm zinc oxide and UiO-66-NH_2_ MOF particles. For cotton, X-ray photoelectron spectroscopy (XPS) shows that plasma treatment partially oxidizes cellulose, with transformation of aliphatic carbons to hydroxyl and carbonyl groups. For nylon, XPS indicates the formation of additional hydroxyl groups and transformation of some amide groups to hydroxyamides. Plasma-treated fabrics and untreated controls were spray-coated with ethanolic suspensions of ZnO or UiO-66-NH_2_ particles, ultrasonicated in ethanol, and air-dried. In some cases, particle-covered fabrics were machine-washed to assess durability. For all three fabrics, scanning electron microscopy (SEM) and XPS demonstrated that plasma treatment significantly enhances the surface concentration of ZnO NPs that remain after ultrasonication. However, machine washing removes most of the ZnO NPs, with minimal differences between untreated and plasma-treated fabrics. In the case of UiO-66-NH_2_, particle sizes of ca. 200 and 500 nm were evaluated, and adhesion was significantly better on NYCO for the smaller particles due to more attachment sites per unit mass; a significant surface concentration of the smaller MOF remains on NYCO even after five wash cycles. To demonstrate the efficacy of the process for treating fabrics to impart chemical protection, permeation times of the nerve agent simulant dimethyl methylphosphonate were compared for bare and MOF-functionalized NYCO. The functionalized fabric exhibited a permeation time that was almost three times greater than for bare, plasma-treated fabric. These results demonstrate the practicality of the process for high-throughput preparation of metal oxide and UiO-66-NH_2_ functionalized NYCO for chemical protection.

## Graphical abstract

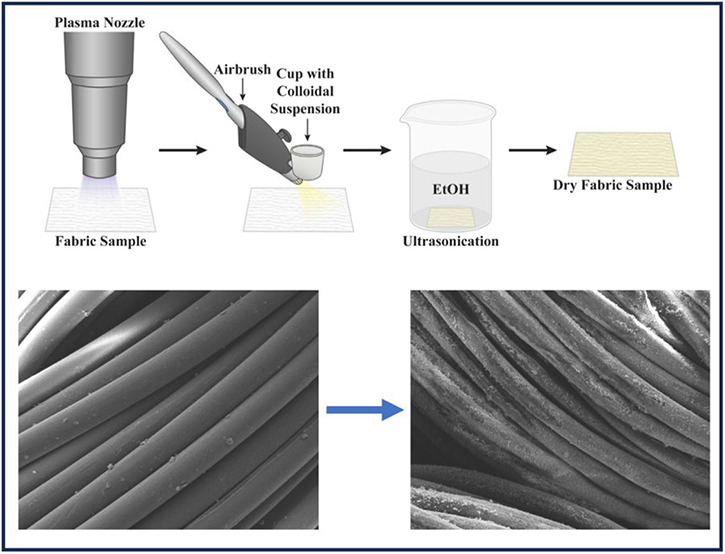

## Introduction

The attachment of nanoparticles to fibers and fabrics is an important area of research, presenting opportunities for advanced materials with a wide range of functionalities. Applications include wide bandgap metal oxide nanoparticles (MONPs) for UV-protective clothing,^1^ additives for flame retardants^2^ and antimicrobials,^1,3,4^ smart textiles for wearable electronics^5^ and for health monitoring.^6^ MONPs and metal–organic framework (MOF) particles have also been incorporated onto fibers and fabrics toward the goal of sorbing and/or degrading hazardous chemicals, including chemical warfare agents.^7–9^ MOFs can also be engineered to capture and neutralize biological agents, including bacteria, viruses and spores, because of their three-dimensional porous structure and metal ions they contain.^10^

These applications have led to the development of particle-fabric attachment methods, including dip-coating,^11^ pad-dry-curing,^12,13^ electrospinning/electrospraying,^1,14^ seeded growth of particles on fabrics,^15^ and atomic layer deposition (ALD).^16–19^ The latter technique has a self-limiting nature and can coat complex surfaces, making it appealing for incorporating nanoparticles on fabrics and fibers. However, its cost-effectiveness and amenability to manufacturing are still being improved.

As specific examples of fabric functionalization with metal oxides or MOFs, Giedraitiené et al.^20^ used sputtering from zinc electrodes in a low temperature oxygen plasma to deposit ZnO NPs onto cotton-based textiles. Bayisa et al.^21^ demonstrated that cotton reacted with l-methionine can covalently bind to Ag NPs and to Ag-ZnO nanocomposites. Rabiei et al.^22^ *in situ* synthesized nanoparticles (e.g., TiO_2_) and MOFs onto cotton-polyester fabrics, and Bunge et al.^15^ seeded cotton fabric with covalently attached Zr, which served as growth sites for UiO-66-NH_2_. Lee et al.^16^ used ALD to form metal oxide “seeds” that then served as nucleation sites for MOF growth.

In recent years, plasma treatment has proven useful for fiber and fabric modification.^23^ Because a plasma contains energetic species, it can form reactive functional groups, even on inert surfaces.^24^ Plasma treatment and other methods of forming reactive functional groups (e.g., ozone treatment) may enable strong adhesion and chemical bonding of nanoparticles.^25^ Open-air plasma treatment, in which vacuum is not required, lends itself to high-throughput manufacturing processes and has been used to increase adhesion to polymers^26^ and between polymer layers,^27^ to stabilize the oxide and semiconducting properties of sputter-processed metal oxide films used for thin film transistor applications,^28^ and to form films with unique wetting properties.^29^

In the present work, the effects of open-air plasma treatment on the surface chemistry of nylon, cotton, and a 50:50 nylon/cotton blend (referred to as “NYCO”) have been investigated toward the goal of enhancing the adhesion of metal oxide and UiO-66-NH_2_ particles, a prototypical Zr-based MOF originally developed at the University of Oslo, on the fabrics. NYCO is an important material because it is comfortable and durable and has widespread use for military uniforms. In this work, it has been found that plasma treatment induces the formation of reactive functional groups that can bond to MONPs and MOFs, and a simple process that is amenable to high-throughput manufacturing has been developed that consists of open-air plasma treatment of a fabric followed by spray coating it with a colloidal suspension of the particles to be deposited. Electron microscopy and X-ray photoelectron spectroscopy (XPS) have been used to assess the effectiveness of this methodology after ultrasonication and durability testing by multiple washing cycles.

## Experimental Section

### Materials

100% cellulose cotton fabric (5.5 oz/sq. yd, plain weave, bleached and mercerized) was purchased from Test Fabrics, Inc. Nylon-6,6 fabric (500 Denier, plain weave) was purchased from Brittany Global Technologies, Inc., and 50:50 nylon–cotton (NYCO) blend fabric (rip-stop weave, 6–6.5 oz/sq. yd) was purchased from Bennettsville Printing, USA. Absolute ethanol from Fisher Scientific was used without further purification. Zinc oxide nanoparticles, with a nominal average particle size of 20 nm were purchased from Nanostructured and Amorphous Materials, Inc. (stock no. 5810HT). Two particle sizes of the Zr-based UiO-66-NH_2_ MOF were obtained from Numat, Inc. Dimethyl methylphosphonate (DMMP), used as an analyte to test the chemical sorption of functionalized fabrics, was purchased from Fisher Scientific.

### Plasma Treatment

Both sides of the fabric samples were subjected to plasma treatment using an open-air plasma system (model FG5001) from PlasmaTreat, Inc., using “breathing air” as the gas source. Use of other gases (e.g., pure oxygen) are not possible with this instrument. The nozzle movement was controlled by a programmable Janome JR3503 Cartesian robot. Plasma exposure was determined by adjusting the distance between the sample and the nozzle, as well as the total residence time beneath the nozzle (part no. 22890). The distance between the plasma gun tip and the sample was set to 5/8 in. The linear speed was ca. 6.0 cm/s, and we estimate the exposure area rate to be ca. 30 cm^2^/s. These parameters were chosen such that the plasma visually uniformly impacted the fabric, but the total plasma exposure, determined by the number of passes of the nozzle over the fabric, was optimized using XPS, as will be discussed shortly. Unless otherwise noted, 20 passes were used, meaning that the nozzle was passed over the fabric 20 times.

### Fabric Functionalization

ZnO nanoparticles or UiO-66-NH_2_ particles were dispersed in absolute ethanol at concentrations of ca. 4 and 3 wt %, respectively, by ultrasonication for 10–15 min. To avoid settling, they were immediately loaded into the “cup” of an airbrush (Testors Aztek) and sprayed onto the fabric of interest. The nozzle of the airbrush was held ca. 3 in. from the fabric and several passes were rapidly made over the area of the fabric such that it was coated uniformly; halfway through the process, it was flipped, and the opposite side was also coated. The fabrics were then sprayed with pure ethanol to remove excess particles. They were then ultrasonicated in ethanol for 15 min to remove loosely bound nanoparticles, using a Branson 2510 ultrasonic cleaner (42 kHz, 100 W). Finally, the fabrics were left to air-dry overnight. Note that this procedure may leave some agglomerated particles still attached to the fibers, as will be discussed.

### Durability Testing

In some cases, functionalized fabrics were washed to assess durability of the coating. Briefly, an AATCC compliant Vortex M6 washing machine set to “normal” was used, with 60 g of standard AATCC detergent (without the brightener) and a water temperature of 30 °C. Each load included 24 pieces of ballast material to simulate a real washing machine, which is standard for the procedure. In this study, either two or five wash cycles were performed on functionalized fabrics. After air drying, the treated fabrics underwent SEM imaging, and XPS analysis was conducted to quantify the presence nanoparticles on the fabric surface. In the case of ZnO NPs, the Zn/C atomic ratio served as a key metric for assessing nanoparticle deposition in this context. For UiO-66-NH_2_, the Zr/C atomic ratio was used as a measure of MOF surface concentration.

### Characterization

Field emission scanning electron microscopy (FE-SEM) and X-ray photoelectron spectroscopy (XPS) were performed on the samples to evaluate the presence and distribution of particles on the fabric surfaces. In the case of FE-SEM, the samples were sputter-coated with a thin layer of gold, and they were imaged using either a JEOL JSM 7401F microscope, using beam energies in the range of 3–7 keV, or a Zeiss Auriga 40 FIB-SEM operating with beam energy of 5 keV. Samples were prepared for XPS by attaching them with double-sided, vacuum compatible tape to the sample stage, and analysis was performed with a SigmaProbe VG Scientific instrument using AlKα X-rays (*h*ν = 1486.6 eV) and a pass energy of 20 eV. The binding energy scale is referenced to the spectroscopic Fermi level, and the spectra were corrected for surface charging by shifting them by the amount necessary to align the aliphatic carbon peak to 285.0 eV. Peak fitting was performed using Avantage Software, with Smart background fitting.

### DMMP Sorption

A custom-built apparatus, employing a 10.6 eV photoionization detector (PID) purchased from Mocon/Ametek (no. 0435–735) and equipped with a “purple” detector module (0–2000 ppm), was used to measure the permeation time of DMMP vapor through MOF-functionalized NYCO. The vapor was generated by bubbling ambient temperature (ca. 20 °C), ultrahigh purity, dry air through liquid DMMP at 400 mL/min, maintained constant by a mass flow controller. This vapor stream was passed over the fabric (which was doubled over on itself) while a computer monitored the PID signal as a function of time. Under these conditions, the concentration of DMMP in the air stream is estimated to be ca. 800 ppm, assuming saturation of the air by DMMP, with a 20 °C vapor pressure of 78.5 Pa.^30^

## Results and Discussion

### Plasma Treatment of Cotton, Nylon and NYCO Fabrics

The effects of open-air plasma treatment on the surface chemistry of cotton, nylon and NYCO were evaluated using XPS, and details of the spectra will be discussed shortly. However, graphs summarizing the O/C and N/C atomic ratios determined by XPS for cotton, nylon and NYCO for 0, 20, and 50 passes are shown in Figure 1. These ratios were calculated by integrating the C 1s, O 1s, and N 1s peaks and dividing the areas, corrected with the transmission function of the spectrometer, by the appropriate sensitivity factors.^31^ As shown in the graphs, there are minimal changes in the atomic ratios due to increasing the number of passes from 20 to 50. Toward the goal of minimizing fiber damage due to excessive plasma exposure and minimizing the plasma treatment time, which would be advantageous for high-throughput processing, 20 passes was chosen for subsequent studies.

**Figure 1:**
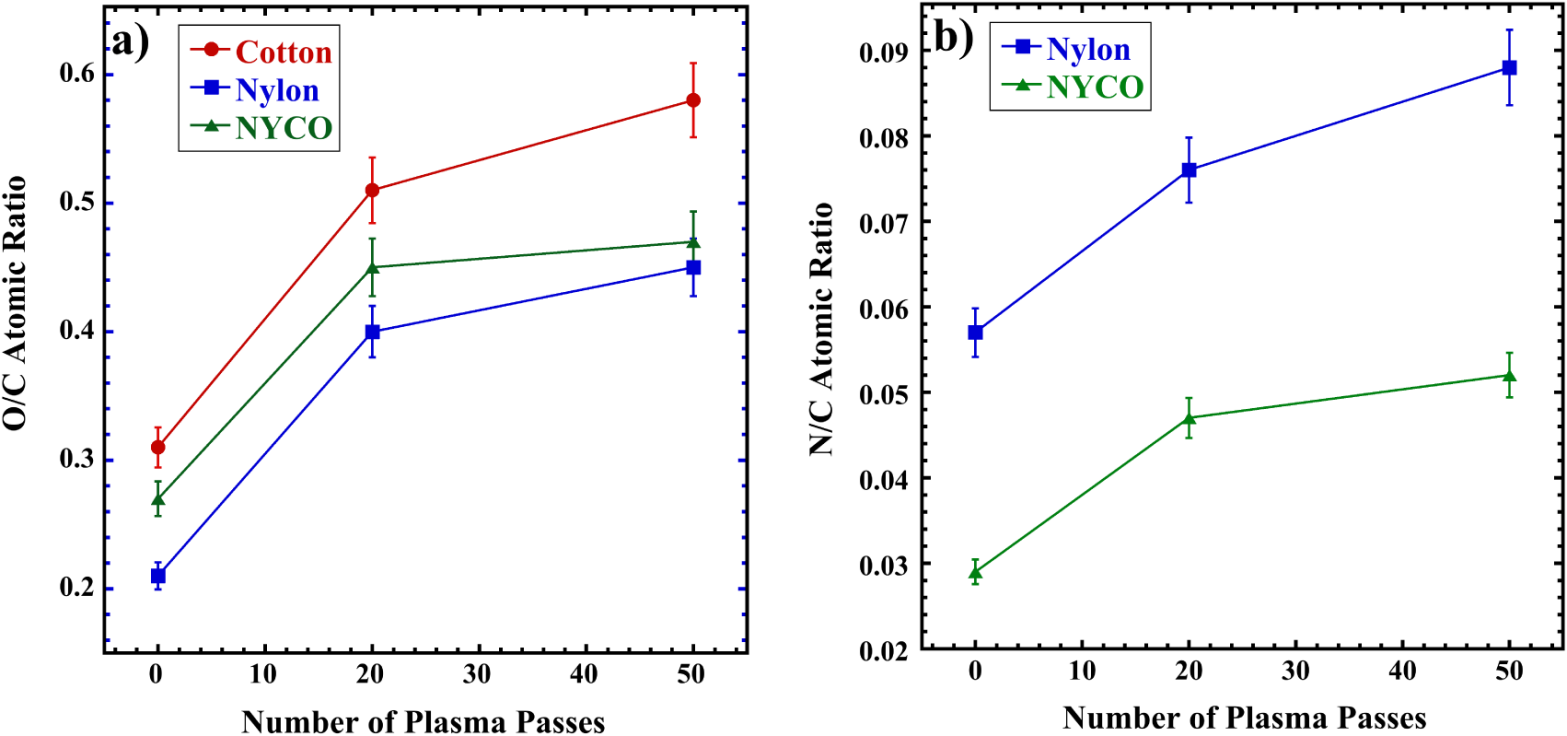
a) O/C atomic ratios for cotton, nylon and NYCO versus number of passes of the open-air plasma treater over the fabric material, as determined by XPS; b) N/C atomic ratios for nylon and NYCO. The error bars were estimated from typical uncertainty in the XPS peak integrations.

Figure 2a shows the C 1s region of cotton before and after open-air plasma treatment. Three C 1s components are present in the untreated cotton sample, at 285.0, 286.7, and 288.4 eV. These are ascribed to aliphatic, C–O–C/C–OH and carbonyl/carboxylate carbon atoms, respectively. While C=O bonds are not present in pure cellulose, they are known to exist in cotton fibers due to their oxidation during pretreatment and noncellulose constituents.^32^ After plasma treatment, the intensities of the higher binding energy components increase relative to the aliphatic carbon atoms, consistent with further oxidation of the cellulose backbone and transformation of aliphatic carbons to hydroxyl and carbonyl groups. There is a slight shift of the carbonyl/carboxylate component toward higher binding energy. Peak fitting of the O 1s XPS spectra (Figure 2b) reveals two components at 531.4 and 532.8 eV. The O 1s level is not as useful in terms of displaying pronounced binding energy shifts due to different functional groups.^33^ However, using information from synthetic polymers,^33^ we ascribe the 531.4 eV component to the oxygen atoms double-bonded to carbon atoms in carboxylate groups and the 532.8 eV component to the oxygen atoms in hydroxyl groups or single-bonded O atoms in carboxylate groups. Both components increase in intensity (relative to carbon) due to plasma treatment, consistent with formation of more carbonyl/carboxylate and hydroxyl groups. The O/C atomic ratio increases from 0.31 to 0.51 due to plasma treatment, and peak fitting results are summarized in Table 1. Note that the “theoretically” expected atomic ratio for pure cellulose is 0.83. The observation of significantly lower O/C XPS atomic ratios indicates the presence of noncellulosic materials on the surface. As shown in Table 1, plasma treatment of cotton causes the percentage of the carbon atoms in an oxidized form (i.e., nonaliphatic) to increase from 27.6 to 42.4%.

**Figure 2:**
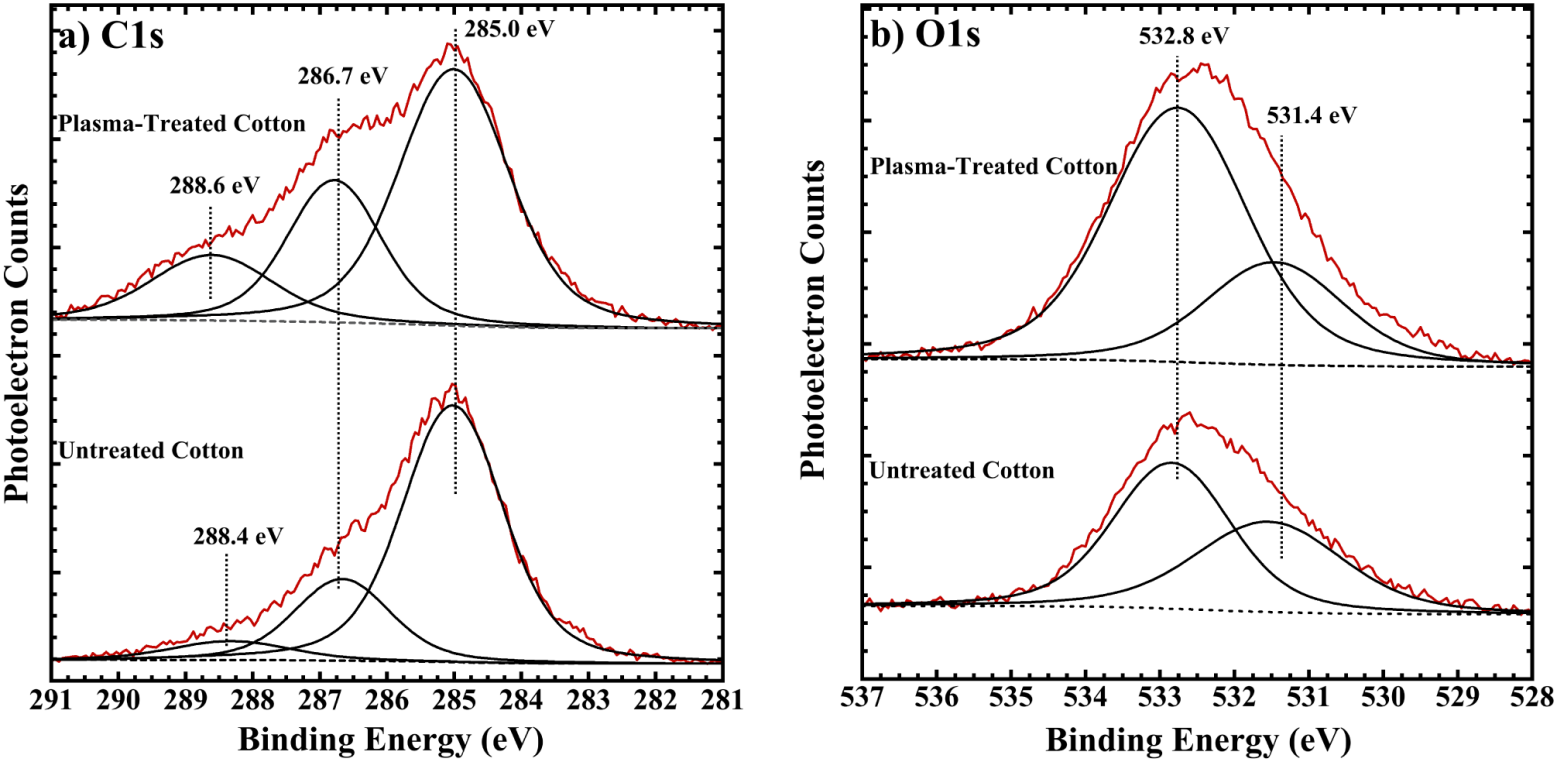
AlK α XPS of the a) C 1s and b) O 1s regions of untreated and plasma-treated cotton fabric. The raw spectra are shown in red, and the fitted components are in black.

**Table 1:** XPS Peak Fitting Results for Untreated and Plasma-Treated Cotton, Nylon and NYCO

Sample	C 1s	O 1s	N 1s	O/C, N/C atomic ratios
Cotton (untreated)	285.0 eV (72.4%)	531.4 eV (37.5%)	N/A	0.31, N/A
	286.7 eV (21.0%)	532.8 eV (62.5%)		
	288.4 eV (6.6%)			
Cotton (20 passes)	285.0 eV (57.6%)	531.4 eV (23.5%)	N/A	0.51, N/A
	286.7 eV (27.3%)	532.8 eV (76.5%)		
	288.6 eV (15.1%)			
Nylon (untreated)	285.0 eV (85.9%)	531.4 eV (72.3%)	399.5 eV (100%)	0.21, 0.057
	287.6 eV (14.1%)	533.1 eV (27.7%)		
Nylon (20 passes)	285.0 eV (71.8%)	531.4 eV (55.6%)	399.5 eV (72.5%)	0.38, 0.076
	286.4 eV (9.2%)	532.6 eV (44.4%)	400.5 eV (18.0%)	
	288.4 eV (19.0%)		408.2 eV (9.5%)	
NYCO (untreated)	285.0 eV (78.7%)	531.2 eV (41.2%)	399.3 eV (100%)	0.27, 0.029
	286.7 eV (15.0%)	532.7 eV (58.8%)		
	288.6 eV (6.3%)			
NYCO (20 passes)	285.0 eV (62.1%)	531.2 eV (28.6%)	399.3 eV (71.4%)	0.45, 0.047
	286.7 eV (24.8%)	532.7 eV (71.4%)	407.0 eV (28.6%)	
	288.6 eV (13.1%)			

Corresponding data for nylon are shown in Figure 3. The C 1s spectrum of untreated nylon contains two components at 285.0 and 287.6 eV, corresponding to the C–C species within the polymer backbone and the C–N/C=O species. These are in a respective ratio of ca. 6:1. Plasma treatment leads to emergence of an additional component at ca. 286.4 eV, which is attributed to carbon atoms attached to newly formed hydroxyl groups, from oxidation of the C–C backbone, and shift of the higher binding energy component to 288.4 eV, likely due to COOH groups. In the case of the O 1s spectrum, there are two components at 531.4 and 533.1 eV, which we ascribe to C=O and hydroxyl groups, with the latter due to surface contamination. Plasma treatment causes the higher binding energy component to increase in intensity relative to the lower binding energy one, consistent with formation of more surface hydroxyl groups, and to shift to 532.6 eV. Included in Figure 3 are corresponding N 1s spectra for nylon. Untreated nylon shows a single N 1s component at 399.5 eV, consistent with the amide group (CONH_2_) in nylon-6,6.^34^ Imide groups, which exhibit a similar binding energy^35^ may also be present. Plasma treatment induces a small component at 400.5 eV, with the nitrogen being attached to a more oxidized carbon, such as for a hydroxyamide. Other authors have observed a similar XPS peak for plasma-treated nylon.^34^ Additionally, a component is observed at 408.2 eV, likely corresponding to nitro (−NO_2_) nitrates or nitrate (−ONO_2_) species following extensive oxidation of the nylon fabric. It is likely that this high binding energy N 1s peak arises from implanted nitrogen from the plasma gas (i.e., air). The theoretical O/C and N/C atomic ratios are both 0.17 for nylon-6,6. Table 1 includes the O/C and N/C atomic ratios, showing that plasma treatment leads to an O/C increase from 0.21 to 0.38 and an increase in N/C from 0.057 to 0.076. The percentage of carbon that is oxidized (i.e., nonaliphatic) increases from 14.1 to 28.2% due to plasma treatment.

**Figure 3:**
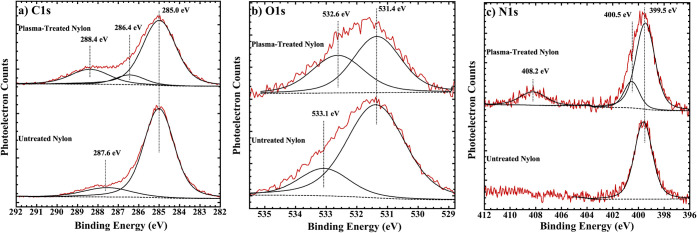
AlK α XPS of the a) C 1s, b) O 1s and c) N 1s regions of untreated and plasma-treated nylon fabric. The raw spectra are shown in red, and the fitted components are in black.

Figure 4 shows a schematic of possible plasma-induced oxidation products of cellulose and nylon-6,6. Plasma treatment of cotton leads to ring opening in the near-surface region of the sample and the formation of carbonyl and carboxylic acid groups. In the case of nylon, hydroxyl groups are formed along with carbonyl groups. It must be noted that the process is nonselective, and a mixture of products is formed. It must also be realized that this is a very idealized schematic, since impurities are present on the fibers, as indicated by the deviation of the measured atomic ratios from their theoretical values. Attenuated total reflectance infrared (ATR-IR) spectroscopy experiments were attempted to gain additional information. However, no significant differences were observed between untreated and plasma-treated fabrics. Unlike XPS, ATR-IR is not a surface sensitive technique, with penetration depths on the order of microns.^36^ The lack of detectable differences suggests that open-air plasma treatment is affecting a very small depth of the near-surface region, perhaps even shallower than with conventional plasma.

**Figure 4:**
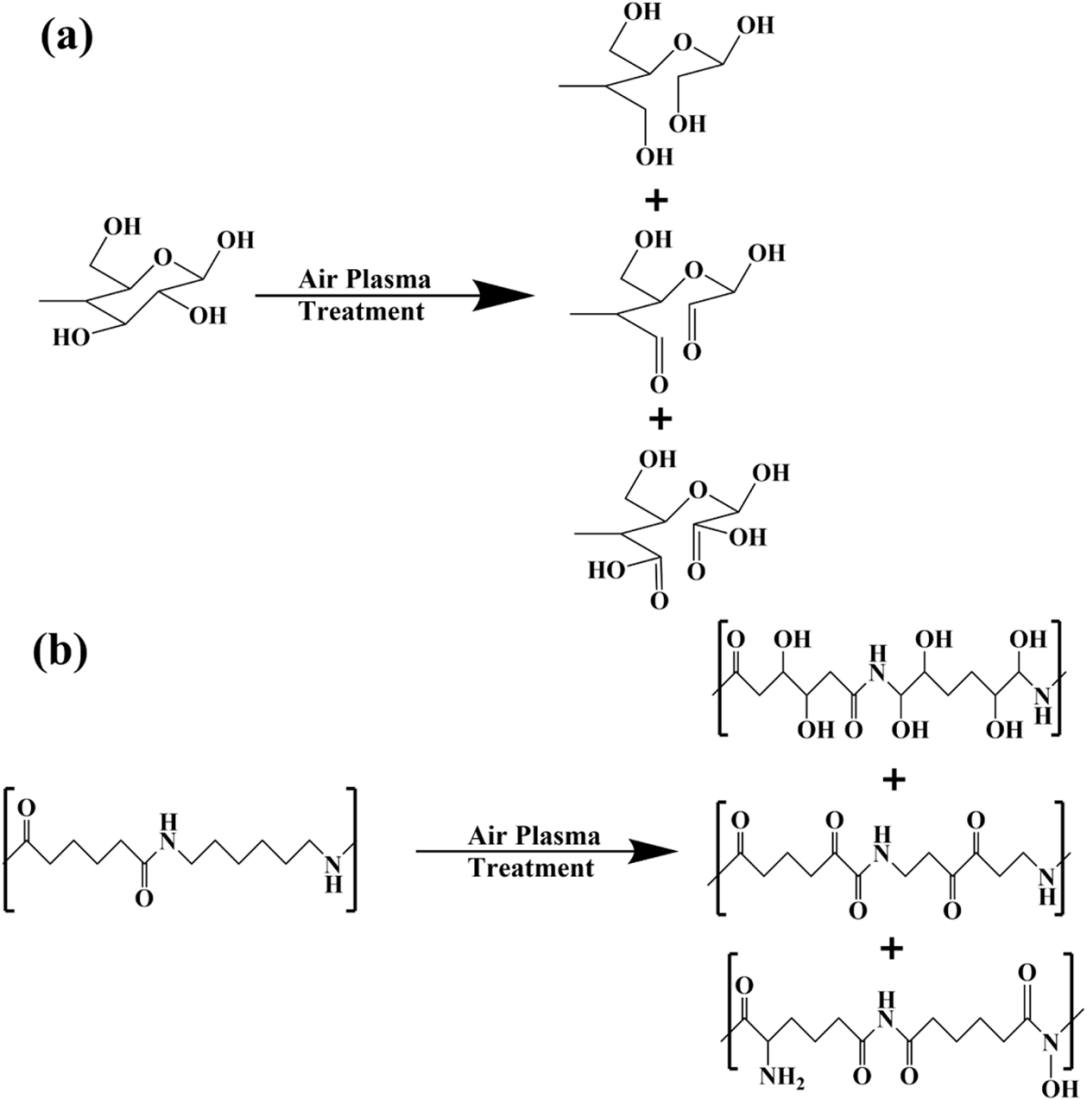
Simplified schematic of the effects of open-air plasma treatment on a) cotton and b) nylon. In the case of cotton, cellulose ring opening and formation of carbonyl and carboxylic acid groups occur. For nylon, plasma-treatment leads to hydroxyl, carbonyl, and possibly imide and hydroxyamide groups.

Unsurprisingly, the C 1s XPS spectrum (Figure 5a) of untreated and plasma-treated NYCO fabrics resemble those for cotton and nylon, with peak components at 285.0 (C–C), 286.7 (C–OH/C–O–C) and 288.6 eV (C=O/COO). Plasma treatment causes the areas of the 286.7 and 288.7 eV components to increase at the expense of the aliphatic carbon component. These findings align with the results for plasma treatment of cotton and nylon (separately) and are consistent with formation of hydroxyl, carbonyl and carboxylic acid groups on the surface of the NYCO fabric.

**Figure 5:**
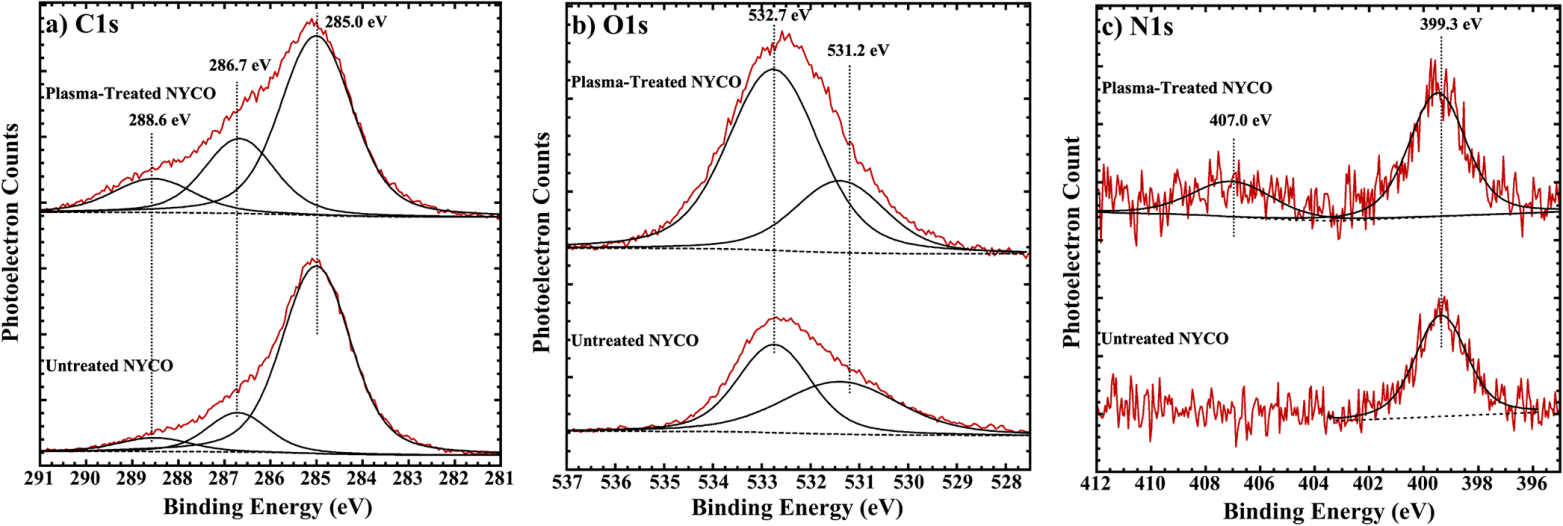
AlK α XPS of the a) C 1s, b) O 1s and c) N 1s regions of untreated and plasma-treated NYCO fabric. The raw spectra are shown in red, and the fitted components are in black.

In Figure 5b of AlKα O 1s XPS, the peak for untreated NYCO fabrics indicates the presence of hydroxyl ligands (−OH) at 532.7 eV, consistent with the C–OH peak detected in the C 1s spectra. A weaker peak associated with carbonyl (C=O) ligands at 531.2 eV is also observed, aligning with the C 1s photoelectron spectra. For the plasma-treated fabrics, the photoelectron peaks associated with hydroxyl (−OH) groups and carbonyl (C=O) groups are also observed at similar binding energies as in the untreated fabrics. The intensities of these peaks and concentration of these groups, however, have appreciably increased.

In Figure 5c, the N 1s XPS spectrum of untreated NYCO shows a single peak at 399.3 eV, corresponding to the amide group seen for nylon. For the plasma-treated NYCO, the peak shifts slightly to 399.5 eV. Because the sample is only 50% nylon, the feature due to hydroxyamide that was observed for pure nylon is not obvious in the spectrum. But, the feature at 407.0 eV, likely resulting from prolonged oxidation of nitrogen species into nitrates or nitro groups, is present. The theoretical O/C and N/C atomic ratios for NYCO are 0.50 and 0.08. As shown in Table 1, plasma treatment causes the O/C value to increase from 0.27 to 0.45, and the N/C value increases from 0.029 to 0.047.

### Functionalization of Fabrics with ZnO Nanoparticles

The surface of ZnO NPs exposed to air are essentially fully hydroxylated. Previous studies on this same lot of ZnO NPs have estimated the surface area and hydroxyl surface density as 17.43 m^2^/g and 4.8 × 10^20^ OH/g, respectively.^37^ The hydroxyl groups facilitate bonding to the surface, likely by a combination of dipole–dipole interactions or covalent bond formation via a condensation reaction between the surface hydroxyl groups of the NPs and carboxylic acid groups on the fiber surface.

Figure 6 shows a schematic of the steps used to plasma treat a fabric sample and deposit nanoparticles on it. ZnO NPs were chosen for initial studies because of their gas/vapor sorption properties^37,38^ and catalytic/photocatalytic applications.^39^ This methodology was applied to ca. 5 in. × 5 in. pieces of cotton, nylon and NYCO using 20 passes under the open-air nozzle, as described earlier. After plasma-treatment, the fabrics were spray-coated, and loosely bound nanoparticles were removed via ultrasonication. It is recognized that in addition to removing loosely bound particles, it is possible that sonication may increase the adhesion of the particles to the fabric that are in direct contact with it by cavitation-mediated dispersion of the NPs.^40^ After sonication and drying, a portion of each sample was analyzed by XPS and/or SEM. Another portion was subjected to wash cycles, as discussed earlier and then air-dried.

**Figure 6:**
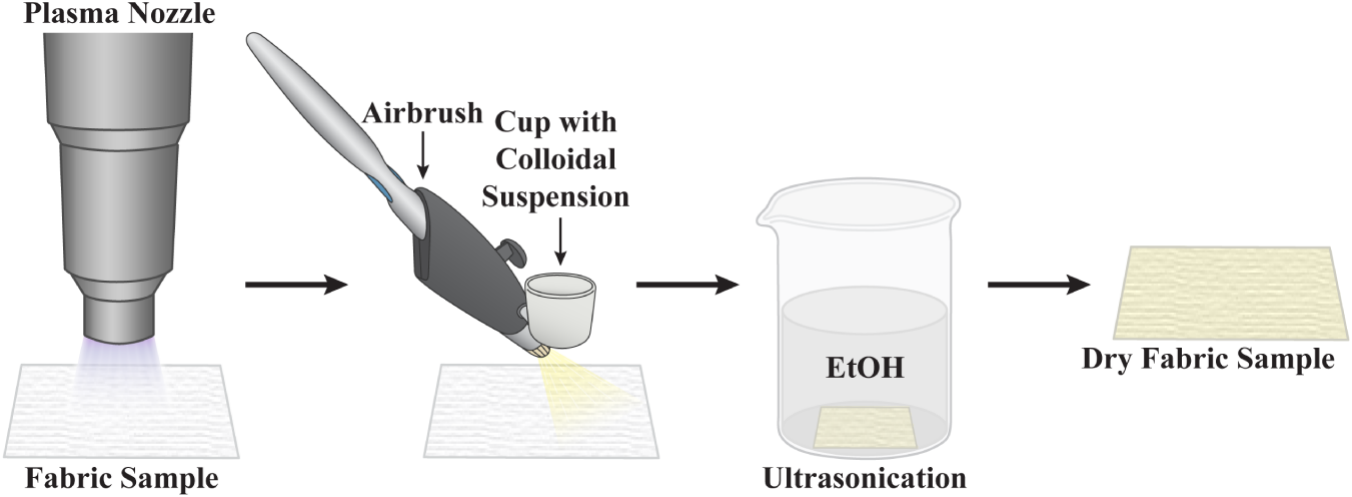
Schematic of the process used to adhere particles to fabric surfaces. Open air plasma treatment of both sides of the fabric is followed by spray-coating with an ethanolic colloidal suspension of the particles. The coated fabric is then ultrasonicated in ethanol to remove loosely bound particles prior to air drying.

Figure 7 shows Zn 2p XPS spectra of cotton, nylon, and NYCO samples corresponding to these treatments. Also included are spectra of control samples that went through the same steps but that were not plasma treated. Greater intensity of the Zn 2p peaks indicates more ZnO adsorption. Included in the figure are Zn/C atomic ratios. The first observation from the data is that some adsorption occurs on all three fabrics, even without plasma treatment. This is especially true for cotton, with the untreated sample having a Zn/C atomic ratio of 0.14. Similar values for untreated nylon and NYCO are 0.079 and 0.070, respectively. The second observation is that plasma treatment enhances ZnO NP adsorption. It is postulated that this occurs because of a condensation reaction between ZnO NP hydroxyl and carboxylic acid groups induced by plasma treatment of the fabric, and dipole–dipole interactions between polar groups on the fabric surfaces and ZnO hydroxyl groups. Cotton, nylon and NYCO exhibit Zn/C atomic ratios of 0.28, 0.17 and 0.22, respectively. The third observation is that two wash cycles remove most of the ZnO NPs, but slightly more remains on the plasma-treated samples than on the untreated controls.

**Figure 7:**
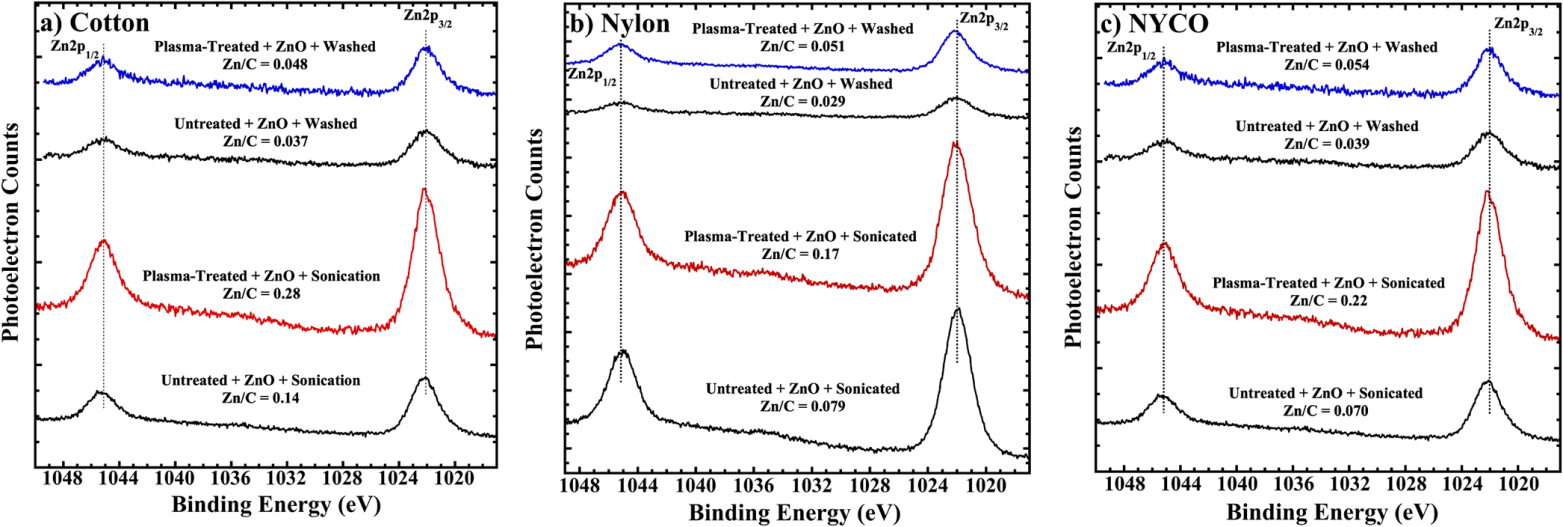
AlK α XPS of the Zn 2p region of untreated and plasma-treated a) cotton, b) nylon, and c) NYCO after they were spray-coated with ZnO NPs and ultrasonicated in ethanol. The top set of spectra for each fabric was then washed to test for durability. The Zn/C atomic ratio for each sample is shown above each spectrum.

Corresponding FE-SEM images are shown in Figure 8. The top row contains images of each of the three untreated fabrics that were spray-coated with ZnO NPs and then ultrasonicated. The second row shows corresponding plasma-treated fabrics that went through an identical procedure. In agreement with the XPS results, plasma treatment enhances the adhesion of ZnO NPs to cotton, nylon and NYCO, with the plasma-treated fabrics exhibiting significantly higher coverage than the untreated controls. The third and fourth rows show images of untreated and plasma treated samples, respectively, that were spray-coated with ZnO, sonicated, and then washed twice. Consistent with XPS, most of the ZnO NPs have been removed in the case of all three fabrics, but only modestly more remain on the plasma-treated fabrics.

**Figure 8:**
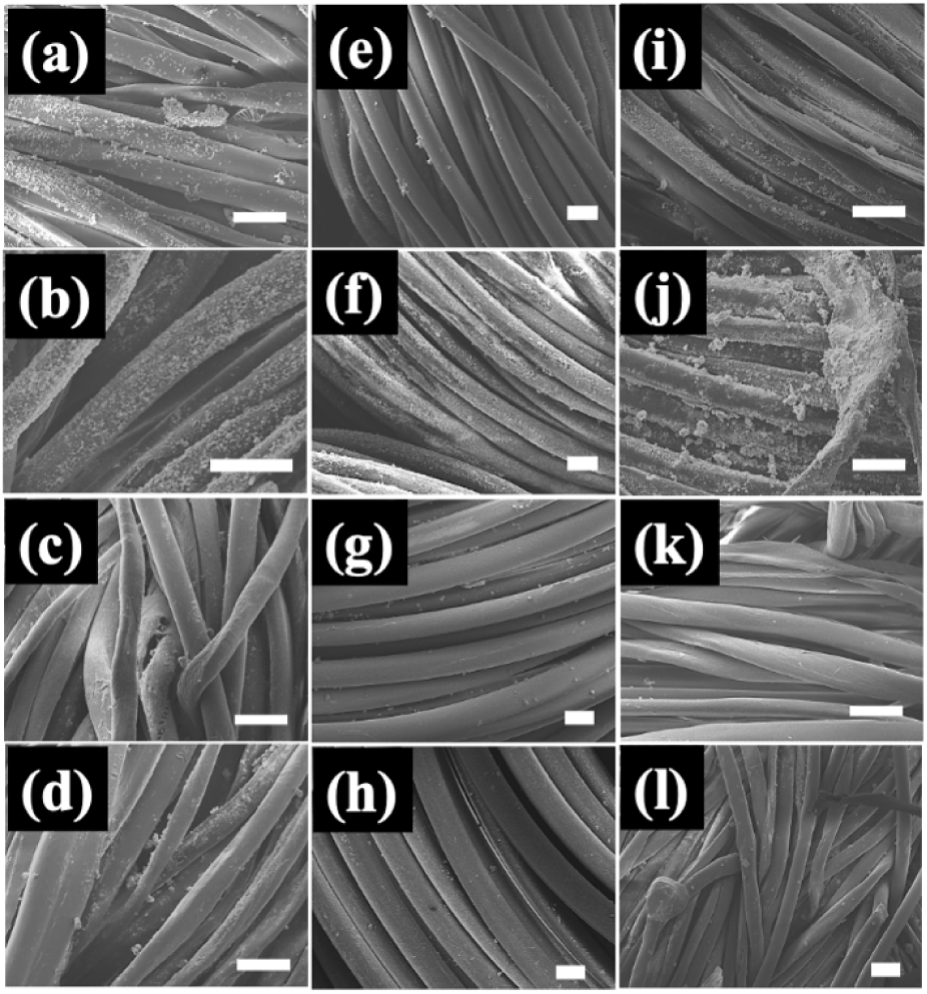
FE-SEM images of fabric samples that were spray-coated with ZnO NPs and then ultrasonicated. The images correspond to a) untreated cotton; b) plasma-treated cotton; c) untreated cotton that was washed after ZnO coating and sonication; d) plasma-treated cotton that was washed after ZnO coating and sonication; e) untreated nylon; f) plasma-treated nylon; g) untreated nylon that was washed after ZnO coating and sonication; h) plasma-treated nylon that was washed after ZnO coating and sonication; i) untreated NYCO; j) plasma-treated NYCO; k) untreated NYCO that was washed after ZnO coating and sonication; l) plasma treated NYCO that was washed after ZnO coating and sonication. The white scale bar in each image corresponds to 10 μm.

### Functionalization of Fabrics with UiO-66-NH_2_

UiO-66-NH_2_, a Zr-based MOF with 2-aminobenzene dicarboxylic acid pendant groups, has been shown to neutralize various hazardous vapors.^41^ This MOF has amine, hydroxyl, and carboxylic acid functional groups that can potentially bond to fabric surfaces. Successfully attaching it to fabrics provides a useful method for creating gas filtration membranes. Two sizes of UiO-66-NH_2_ particles were investigated, with sizes of ca. 230 ± 40 and 520 ± 80 nm, as shown in the SEM images of Figure 9. As will be shown, adhesion of the larger size MOF is inferior to the smaller one. Because of this, most experiments were performed on the smaller size UiO-66-NH_2_. Figure 10 displays FE-SEM images of untreated and plasma-treated cotton, nylon and NYCO samples following spray coating with the smaller MOF, ultrasonication, washing twice and then air-drying. Washing is a rigorous test of their durability and removes loosely bound particles. For each pair of FE-SEM images, the one on the left exhibits significantly more MOF particles than the one on the right that corresponds to the untreated sample. There are some bare spots on the plasma-treated fibers, but the majority of the coating on the fiber surfaces has endured the washing cycles.

**Figure 9:**
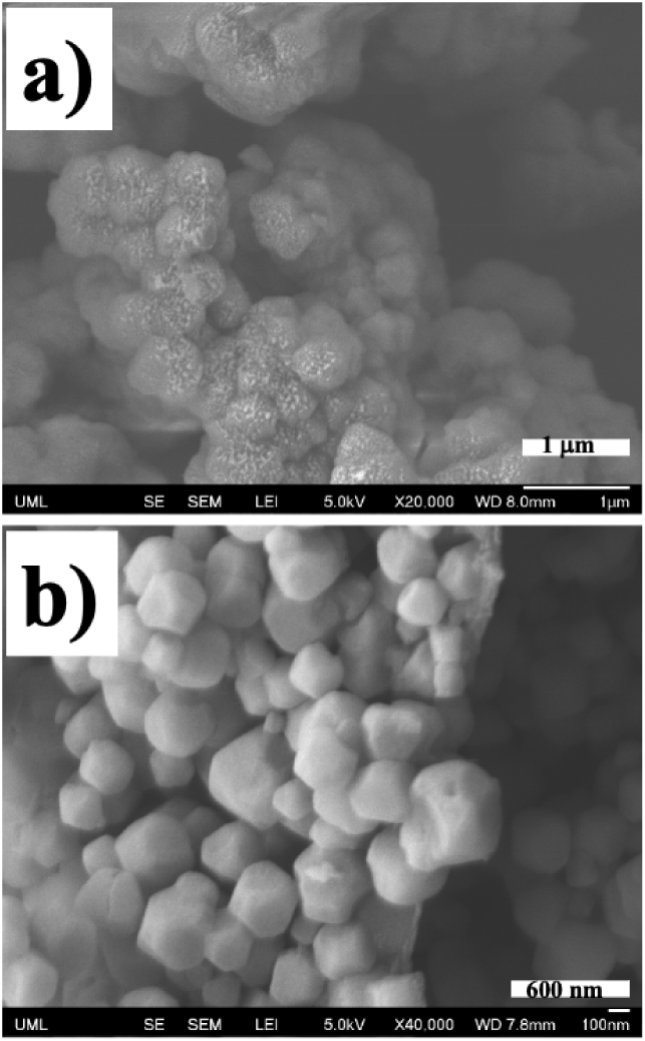
FE-SEM images of the two UiO-66-NH_2_ MOF samples used for this study. The larger size MOF is shown in a), and the smaller size MOF is shown in b). The white scale bar in a) corresponds to 1 μm, while the one in b) corresponds to 600 nm.

**Figure 10:**
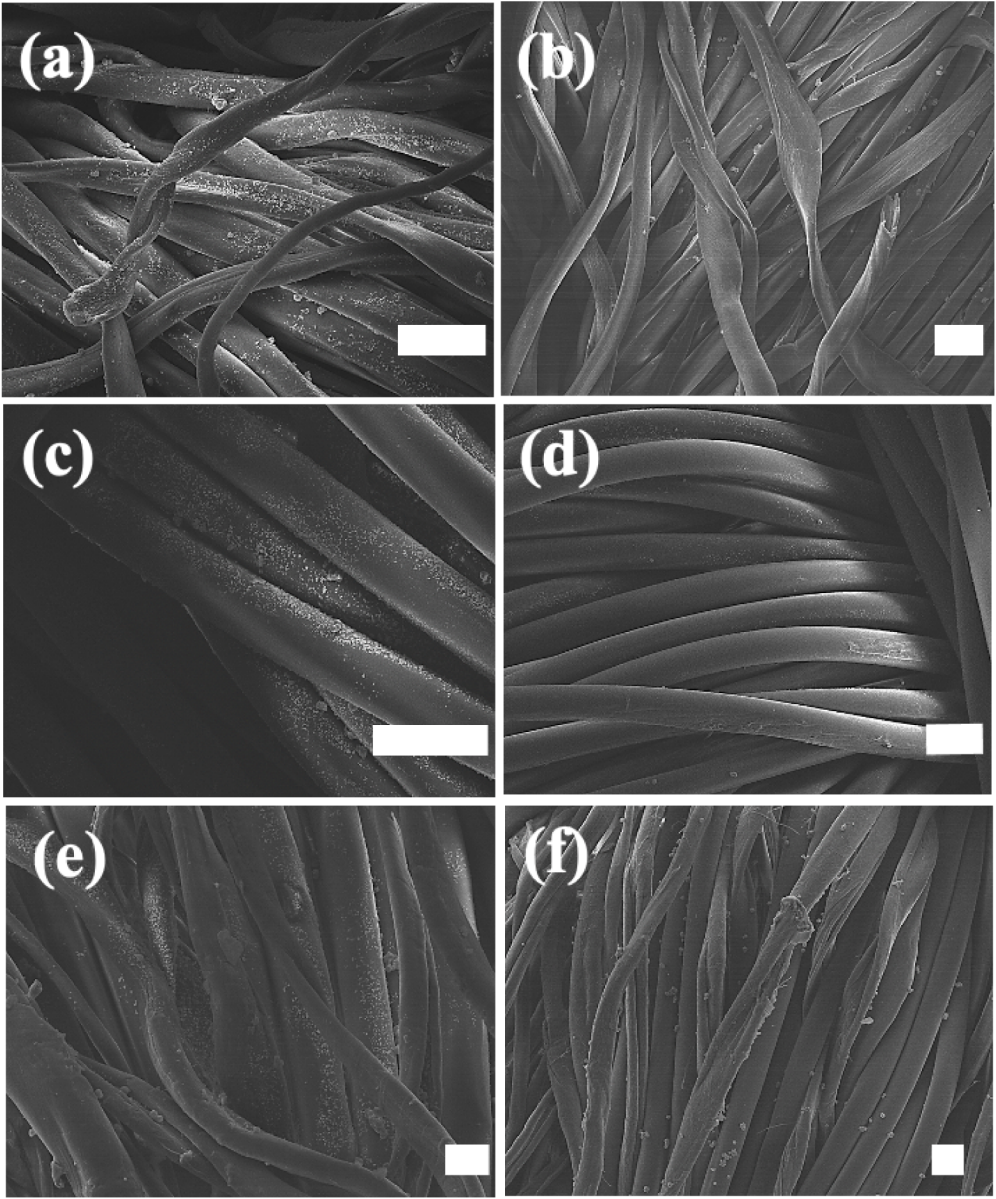
FE-SEM images of fabric samples that were spray-coated with the smaller size UiO-66-NH_2_, ultrasonicated, washed twice and then air-dried. The images correspond to a) plasma-treated cotton; b) untreated cotton; c) plasma-treated nylon; d) untreated nylon; e) plasma-treated NYCO; and f) untreated NYCO. The white scale bar in each image corresponds to ca. 10 μm, except for f), in which it corresponds to 15 μm.

XPS is a better technique than SEM to evaluate relative coverage because it generally interrogates a larger region of the sample, providing an average value. Because UiO-66-NH_2_ contains ca. 3 at. % of Zr, the Zr/C atomic ratio can be used as a measure of the amount of UiO-66-NH_2_ attached to the fabrics. Figure 11 displays Zr 3d XPS for untreated and plasma-treated NYCO functionalized with “small” and “large” sizes of UiO-66-NH_2_, and the corresponding spectra after washing. The Zr/C atomic ratio is included above each spectrum. Similar to what was observed for ZnO NPs, UiO-66-NH_2_ adhesion is enhanced by plasma treatment, with ca. 2.2 and 27 times higher large and small MOF concentration, respectively, remaining on the NYCO after ultrasonication, compared to untreated fabrics. Poor adhesion in the case of the large size UiO-66-NH_2_ is likely due to the smaller surface area-to-volume ratio of the large particles and fewer attachments sites per mass of particle.

**Figure 11:**
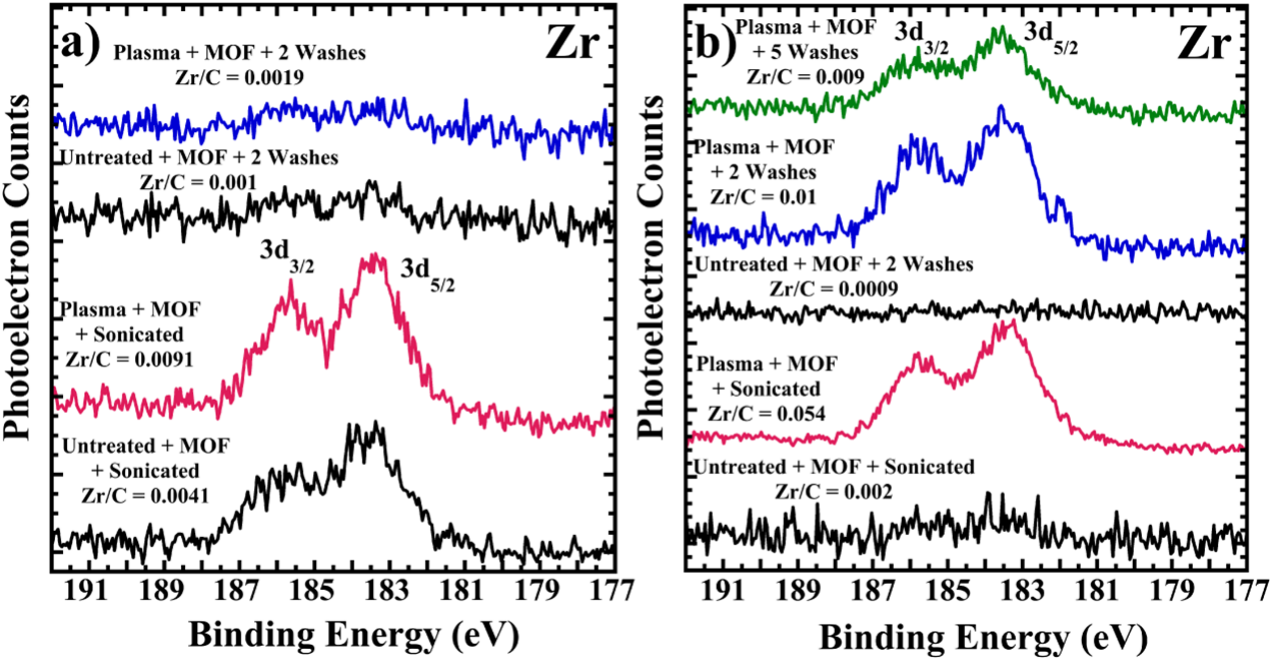
AlK α XPS of the Zr 3d region of untreated and plasma-treated NYCO that was then spray coated with a) large and b) small size MOF. Samples of the fabrics were sonicated and air-dried, and a portion of those was also washed either 2 or 5 times (as indicated) and then air-dried prior to analysis. The Zr/C atomic ratios are given in the figures. In the case of the larger size MOF, most of it was removed by washing twice, as indicated by the negligible Zr 3d signal.

While two wash cycles remove most of the MOF, especially in the case of the larger size UiO-66-NH_2_, substantially more remains on the plasma-treated NYCO (Zr/C = 0.01 vs 0.0009) for the smaller MOF. Furthermore, five wash cycles only decrease the Zr/C ratio by a small amount compared to two cycles, with the Zr/C atomic ratio decreasing from 0.01 to 0.009. The estimated experimental error in the Zr/C atomic ratio is 10%, implying that the Zr/C atomic ratios are the same within experimental error for two and five wash cycles.

SEM images dramatically illustrate the effect of five washes on untreated and plasma-treated NYCO samples that were spray-coated with the smaller MOF. Prior to washing, but after ultrasonication (Figure 12a), MOF particles are present on the untreated fiber surface but not uniformly covering it. Five wash cycles remove most of the particles (Figure 12b). However, for the plasma-treated NYCO sample, the fibers are fully, densely covered after ultrasonication but prior to washing (Figure 12c). This suggests that ultrasonication does not fully remove agglomerated particles. Five wash cycles remove most of the multilayers of particles but leave the fibers fully covered (Figure 12d). These images are in general agreement with the XPS results.

**Figure 12:**
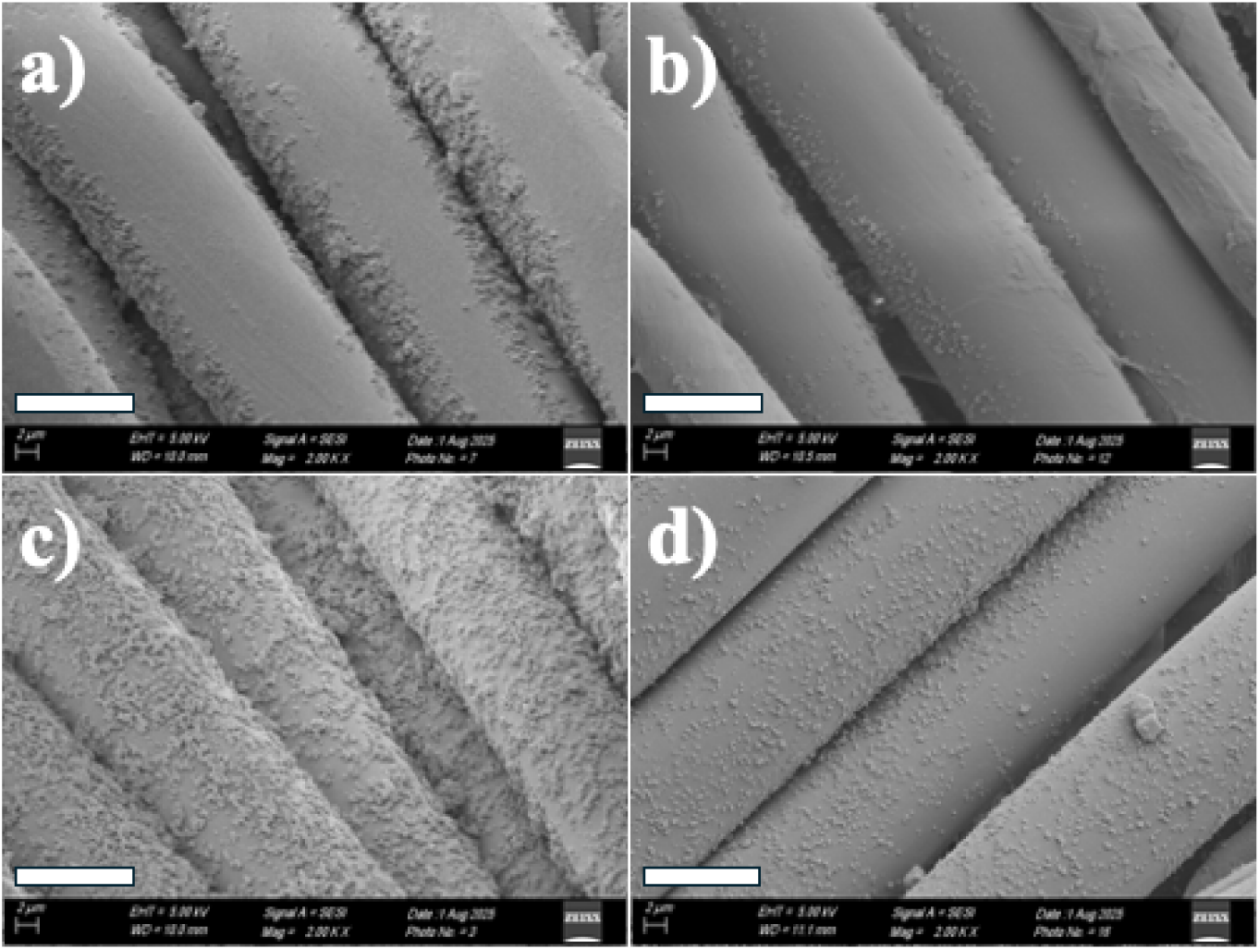
SEM images of NYCO fibers coated with “small” UiO-66-NH_2_ MOF. a) Fibers that were not plasma treated prior to spray-coating with UiO-66-NH_2_ and ultrasonicating; b) the same sample after 5 washes; c) plasma-treated NYCO after spray-coating and ultrasonicating; d) plasma-treated NYCO after spray-coating, ultrasonicating and 5 wash cycles. The scale bar is 12 μm.

It is interesting that the small-size UiO-66-NH_2_ functionalized NYCO is more durable than the ZnO functionalized samples. Adhesion of ZnO NPs to fibers is likely via a combination of covalent bonds, due to a condensation reaction between the ZnO hydroxyl groups and carboxylic acid groups of the plasma-treated cotton (i.e., formation of ester bonds), and dipole–dipole interactions between the ZnO hydroxyl groups and the polar groups of the cotton and nylon fibers. None of these bonds are expected to withstand the alkaline conditions present during washing. While UiO-66-NH_2_ has abundant functional groups (OH, COOH and NH_2_) to form ester and imine bonds and participate in dipole–dipole interaction with the treated fibers, these bonds are also subject to hydrolysis in an alkaline environment. However, the surface area of UiO-66-NH_2_ is in the range of 300–1000 m^2^/g,^42^ and is much higher than that of the ZnO NPs (17.43 m^2^/g). The higher surface area and abundance of surface polar groups on the MOF is likely responsible for the greater durability of the smaller size MOF on NYCO compared to ZnO NPs. In a sense, there is a “chelation effect” in which many bonds would need to be broken simultaneously for the smaller MOF particles to be removed.

UiO-66-NH_2_ has been shown to be active toward capture/neutralization of the nerve agent simulant dimethyl methylphosphonate (DMMP).^41^ Because of this, it seemed important to demonstrate the efficacy of UiO-66-NH_2_ functionalized NYCO toward DMMP capture. The apparatus shown in Figure 13 was constructed, and the permeation times for DMMP to reach the PID was measured for untreated, plasma-treated, and plasma-treated NYCO functionalized with the small size MOF (ultrasonicated and dried, but not washed). Permeation curves are included in the figure. The permeation times for the untreated, plasma-treated, and plasma-treated NYCO functionalized with UiO-66-NH_2_ are 30, 40, and 110 s, respectively. These results indicate that plasma treatment of NYCO causes only a small increase in the permeation time. This is likely the result of increased surface roughness and more polar functional groups on the NYCO surface. However, the DMMP permeation time of plasma-treated NYCO increases dramatically when it is functionalized with MOF (from 40 to 110 s). The increased time is due to sorption of the DMMP vapor by the UiO-66-NH_2_ particles, in accord with results showing activity of this MOF to DMMP sorption.^41^ To test whether DMMP was trapped or decomposed by the MOF-functionalized fabric, and in a separate experiment, a quadrupole mass spectrometer was used to monitor the gas stream exiting the MOF-covered fabric. Phosphonic acid and methyl phosphonic acid are known DMMP hydrolysis products,^43,44^ with their cations having mass-to-charge ratios of 82 and 96, respectively. Absence of these signals in the gas stream exiting the functionalized fabric suggests that the DMMP is not decomposed but trapped intact by the MOF, at least in the case of dry air carrier gas.

**Figure 13:**
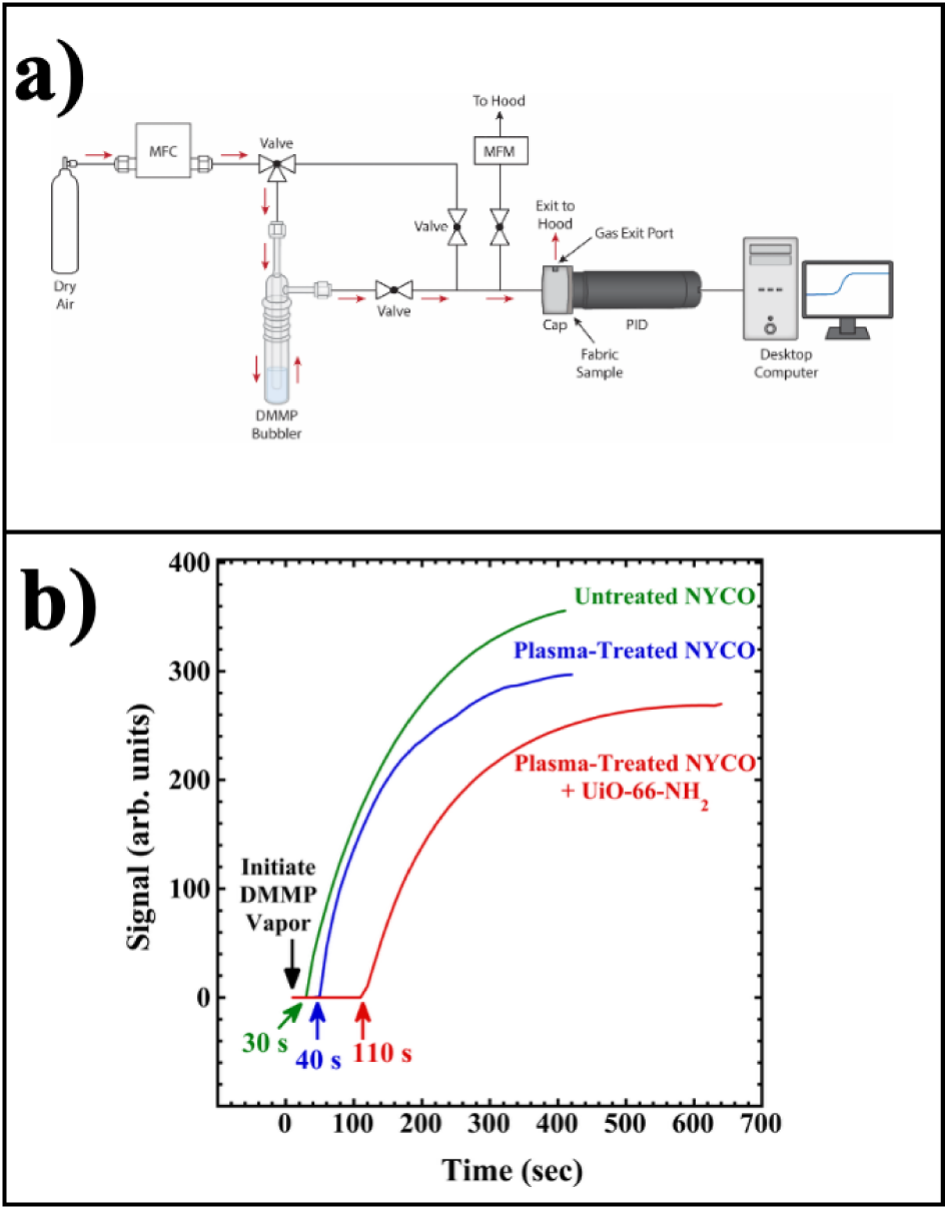
a) Custom-built apparatus for measuring the permeation time for DMMP vapor through fabrics. Air flow is controlled by a mass flow controller (MFC), and the exiting gas flow rate is monitored by a mass flow meter (MFM). The fabric sample is doubled over on itself and inserted in front of the photoionization detector (PID), with the air/DMMP mixture flowing over the fabric before exiting to a fume hood. b) DMMP photoionization signal versus time for untreated NYCO, plasma-treated NYCO, and plasma-treated NYCO that was functionalized with the smaller size MOF, ultrasonicated and air-dried. Onsets of DMMP detection are indicated by arrows.

## Conclusions

The present results demonstrate that open-air plasma treatment, which lends itself to roll-to-roll processing and efficient production, is a viable method to increase the adhesion of nanoparticles onto cotton, nylon and NYCO fabrics. The latter is especially significant because of its use for military and workforce garments. XPS demonstrated that ZnO and UiO-66-NH_2_ may be successfully attached with reasonable stability to plasma-treated cotton, nylon and NYCO. However, ZnO attachment does survive machine washing well. The smaller size UiO-66-NH_2_ (ca. 220 nm), however, attaches extremely well to NYCO and can withstand at least five wash cycles, leaving UiO-66-NH_2_ particles on the fibers. This is likely because of the abundance of polar groups on the MOF and its very high surface area. While this methodology, unlike some others such as ALD, does not form arbitrarily thick layers of MONPs or MOFs, it may be useful for niche applications in which thin layers (or a monolayer) of reactive particles are adequate for filtration applications.
